# Supplementary material for: The causal relationship between the human gut microbiota and pyogenic arthritis: a Mendelian randomization study
Source: Front Cell Infect Microbiol. 2024 Nov 26;14:1452480. doi: 10.3389/fcimb.2024.1452480 (PMC11629706; doi:10.3389/fcimb.2024.1452480)
Supplement: Supplementary file 1 [file DataSheet1.zip › mendelian test/ebi-a-GCST90027518.csv_leave_one_out.pdf]

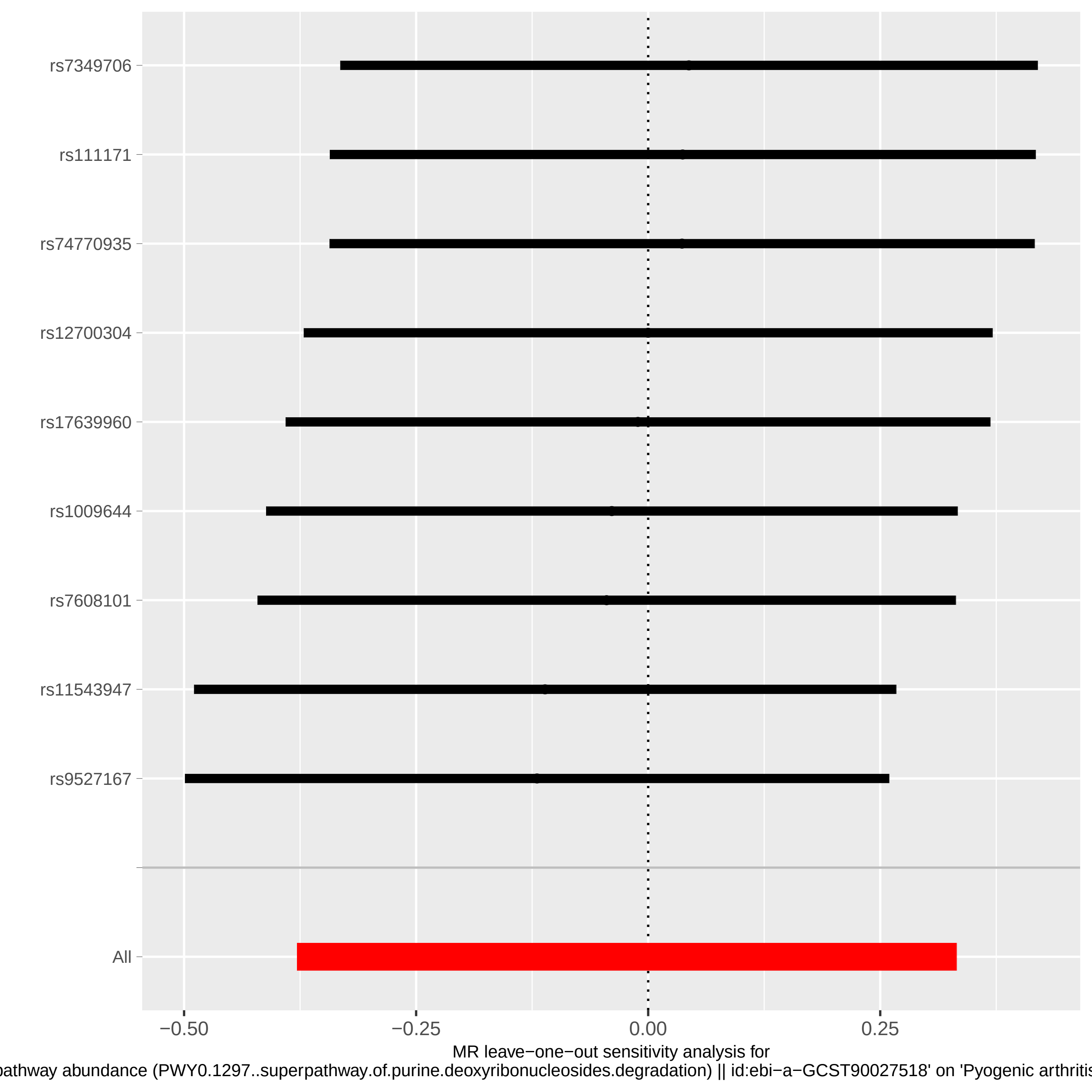
rs7349706
rs111171
rs74770935
rs12700304
rs17639960
rs1009644
rs7608101
rs11543947
rs9527167
All
−0.50
−0.25
0.00
0.25
MR leave−one−out sensitivity analysis for
pathway abundance (PWY0.1297..superpathway.of.purine.deoxyribonucleosides.degradation) || id:ebi−a−GCST90027518' on 'Pyogenic arthritis
